# Consumption Localities

**Published:** 1876-07

**Authors:** 


### CONSUMPTION LOCALITIES.

From information derived from many portions of the United States, the conclusion is clearly warranted, that all low, damp places facilitate the development of consumption of the lungs.

On the other hand, high and airy situations are largely exempted from this malady.

Mary was sent out for recreation for half-an-hour, as was the daily custom. Not knowing any better, she sat on a stone step in the sun, and daily did so. Thus, coming from a warm school-room, and remaining still in the open air, until most thoroughly chilled, she acquired a permanent cough. She sleeps in the churchyard now. How many bright hopes have been blasted; how many an only child has been sent to an early grave, by ignorant, careless, and incompetent teachers, is frightful to think of.

